# The Improvement of Teaching Ideological and Political Theory Courses in Universities Based on Immersive Media Technology

**DOI:** 10.3389/fpsyg.2022.877288

**Published:** 2022-04-29

**Authors:** Li Su, Mengzuo Li

**Affiliations:** School of Marxism, Liaoning University, Shenyang, China

**Keywords:** effectiveness, ideological and political theory courses, colleges and universities, immersive media technology, teaching

## Abstract

This paper focuses on the characteristics of immersive media technology and the advantages, problems and solutions in applying this technology to improve the teaching effectiveness of ideological and political theory courses in colleges and universities. Firstly, it introduces the current development and characteristics of immersive media technology. Secondly, it analyzed the outstanding advantages of immersive media technology in teaching from the following perspectives: virtual reality and augmented reality; sensory stimulation and emotional experience; and human-computer interaction and self-harmony. Thirdly, it puts forward the ways to improve the teaching effectiveness based on immersive media technology from the aspects of theoretical study, history study, and practical study of ideological and political theory courses in colleges and universities. Finally, it discusses the problems in applying immersive media technology to teaching the above courses and puts forward some solutions.

## The Concept and Characteristics of Immersive Media Technology

### Immersive Media Technology

The concept of immersion originates from the immersion theory put forward by American psychologist Mihaly Csikszentmihalyi. According to this theory, immersion refers to the pleasure people may experience when they complete devotion to and concentration on ongoing activities and situations (Mihaly, 2017). When people are engaged in an activity that can attract their full attention and if the challenge coming with it matches their ability, they will be in a mental state of heightened focus and get along with the activity smoothly and efficiently, with all irrelevant perceptions filtered out and time standing still for them. This psychological experience is the state of immersion. We have immersive media when the above theory is applied to human-computer interactive media.

In *Universal Principles of Design*, the word “immersion” is interpreted as a kind of extreme concentration, an utter forgetfulness of the natural world around (William, 2010). This enables people to focus on the target situation created by the designer and thus feel pleasure and contentment. To experience the state of immersion requires two prerequisites. One is the experience atmosphere. Being immersed in something means one is literally immersed in all the relevant information (as one is immersed in water while diving) with all the irrelevant things staved off. Physically and psychologically, we perceive the external world through sensory stimulation, including a series of sensory stimuli such as sight, hearing, touch, smell, and taste. The outside world stimulates people’s senses through the joint action of these sensory channels, thus enabling people to form a perception of their environment and have a three-dimensional and all-around cognition of the outside world. Therefore, the other prerequisite is: in order to realize the state of immersion, it is necessary to create an immersive situation in which people’s senses can be integrated into the content provided by the immersive media (Chen, 2020).

All audio and video technology that enables people to immerse themselves can be called immersive media. For example, a video call can make the caller have the same experience as talking face to face with a person who is far away. Another example is watching a movie on a high definition TV set. Though the viewer may have the feeling of coming to the scene presented in the movie, the immersion experience that these scenes can provide is not complete and thorough (Fu Y. 2020; Fu Y. Y. 2020).

One of the essential ways to achieve deeper immersion hinges on the isolation degree of the immersion atmosphere and to what extent one can stay undisturbed by external stimuli. Special immersive terminal equipment, such as a VR helmet, will produce a powerful sense of visual immersion. The immersive experience provided by VR technology makes use of the input oneness of human visual senses to create an experienced atmosphere that shields other irrelevant external visual stimuli, so that users can be completely immersed in the virtual world and forget about the real world. Another factor that affects the depth of immersion experience is content design. The balance between ability and challenge is one of the first conditions for an individual to realize the immersion experience (Mihaly, 2009). When completing a specific task, one can fully realize whether there is a match between his existing skills and the challenges. Immersive experiences are most easily achieved when the individual has a clear goal and can perceive the skill level to complete the challenge. For example, the most popular video games tend to be ones with a specific challenge that the players themselves are capable of handling. If a game is too difficult, players will often feel anxious and frustrated after failed attempts rather than have fun and satisfaction that the game itself should give, and as a result, they will inevitably give up in the end. If it is too easy, players are also likely to give up the game because of boredom and get immersed in it. Therefore, in the immersive experience, designing challenges for the experiencers, reasonably setting the balance between challenges and skills, and giving immediate feedback on the completion of the challenges will help the experiencers to build up the immersive experience.

Technically, when the immersion theory is extended to human-computer interaction, AR (augmented reality) technology and VR (virtual reality) technology are developed to provide a deep immersion experience. The emergence of AR and VR has enabled people to find ways to use technology to describe the world in immersive media better. Relying on the industrialization process of computer 3D modeling, near-eye display, surround sound, force feedback vibration, micro-sensors and other technologies, immersive media will achieve the vividness of vision, hearing, touch, and smell and smoothness of interaction in the real world. Under such conditions, the users will enter a mode of shared experience in which their consciousness is concentrated in a small range, other irrelevant perceptions and thoughts being filtered, and only specific goals and explicit feedback being responded to. This produces a sense of control and affirmation of their own ability. The application of the above-mentioned technologies and devices to media effects immersive media technology.

### Application Prospect of Immersive Media to Improving Teaching Effectiveness in Education Field

Immersive media, as a brand-new media that combines psychology, acoustics, optics, and computer virtual technology, has been applied in various fields at present. The most typical one is the cultural tourism industry. Using immersive media technology and AR, VR and somatosensory interactive devices, a virtual environment is created according to historical facts, ideas or plots, so that participants can immerse themselves in the virtual environment to see, feel and interact, and their experience is significantly improved. Another broader stage for immersive media technology to exert its application value is the field of education. When the application of immersive media technology can be consistent with the teaching theory, the correct design and appropriate arrangement will provide strong support for the expansion and deepening of the course contents and the improvement and optimization of learning effectiveness. This is almost beyond traditional teaching methods.

The wave of educational informatization has unprecedentedly highlighted the power of education. By integrating educational informatization, innovating teaching modes, enriching teaching means and improving teaching effectiveness, immersive media technology is currently one of the most popular innovative means in the field of education reform in the world. Whether it is in the case of school education or social education, the contribution of immersive media technology is prominent. At present, quite a few universities in China have introduced immersive media technology to classroom teaching to improve the teaching effectiveness of some courses previously taught poorly in the traditional teaching model. In particular, the application of immersive media technology will effectively diversify and reify the teaching of ideological and political theory courses, which are the essential courses to implement the fundamental task of building up moral integrity and cultivating talents of virtues in the whole process of higher education. It can effectively help students to reduce cognitive load, stay motivated, improve practical ability, and cultivate positive emotions, thus achieving the desired teaching effectiveness.

Chinese President Xi Jinping once emphasized that “we should use new media and new technologies to conduct our work vigorously, promote the high integration of traditional advantages of ideological and political education with information technology, and enhance the sense of the times and attraction” (Cao, 2017). In order to dynamize the teaching of ideological and political theory courses in colleges and universities, it is essential to innovate the teaching contents and have more effective educational carriers and forms. Immersive media technology is currently representative of cutting-edge media technology. It can be applied to improve the teaching effectiveness of ideological and political theory courses in colleges and universities by enhancing the sense of the times and attraction. With further development and maturity of computer virtual technology, sensor technology and network technology, the immersive media technology integrated into educational informatization is bound to flourish. Further integration between education and this technology is well on the way. It is bound to provide more convenient and efficient learning conditions for teaching ideological and political theory courses in colleges and universities and then cultivate more high-quality talents for our society.

## The Prominent Advantages of Immersive Media in Improving the Teaching Effectiveness of Ideological and Political Theory Courses in Colleges and Universities

Immersive media has outstanding advantages in solving the problems of monotony and abstraction in the traditional teaching of ideological and political theory courses, helping students to reduce cognitive load, stay focused and motivated, and enhancing practical ability and team spirit. The tapping advantages of immersive media technology will make teaching ideological and political theory courses in colleges and universities yield twice the result with half the effort.

In order to verify and evaluate the advantages of immersive media in improving the teaching effectiveness of ideological and political theory courses in colleges and universities, experiments have been carried out among undergraduates in Liaoning University. Subjects participated in the experiment through voluntary registration. After excluding those who already knew about the experiment content, 60 undergraduates from Liaoning University were recruited as subjects. During the experiment, 240 valid questionnaires were collected. See Table 1 for the specific information of experimental subjects.

**Table 1:** Information form of experiment subjects.

		Total number of people	Proportion
Academic degree	Undergraduate	60	100%
Age bracket	18–20 years old	60	100%
Gender	Male	24	40%
	Female	36	60%
Specialized subject	There are seven majors involved.		

The materials used in the experiment included traditional teaching media and immersion teaching media. Traditional teaching media included PPT slides (including text and static pictures) based on Section 2 of Chapter 5 in *Outline of Modern Chinese History*, a textbook for university ideological and political courses, and the documentary *Long March* filmed by China Central Television. Immersive teaching media included the immersive virtual simulation course system “Stay Confident on the Long March in the New Era—Decoding the Long March Spirit” produced by Liaoning University, which could give subjects an overview of the Long March through time and space in the virtual Long March Memorial Hall. In addition, there was the VR interactive simulation experience system “Crossing Jinsha River” developed by Peijing Technology. The experiencer would become a boatman and help the Red Army main force to cross the torrential Jinsha River through human-computer interaction.

The 7-day experiment was conducted in the conference room and the multi-functional classroom of Marxism College of Liaoning University. The conference room had a big screen for PPT and video. The multi-functional classroom was equipped with 10 computers for VR equipment experience. During the experiment, 60 students were evenly divided into Group 1 and Group 2. Group 1 only used traditional teaching media to study, while Group 2 adopted immersion teaching media. The 30 students in Group 1 were taught the content of the Long March in the second section of Chapter 5 of *Outline of Modern Chinese History* by teachers in the conference room in one class, and in the other class, they watched the teaching video *Long March* (excerpt) and then filled out the questionnaire. The 30 students in Group 2 listened to the teachers in the conference room together with Group 1 in the first class. Then, in the second class, they went to the multi-functional classroom where they were subdivided into three groups of 10 students and continued to study using immersive teaching media. They watched videos, wore VR equipment, and filled out relevant questionnaires. After data collection, we used EXCEL for data statistics and analysis.

### The Blending of Virtual Reality and Augmented Reality Stimulates Excellent Attention Restoration Ability

The application of immersive media in promoting education and teaching practice is mainly based on VR, AR, and other technical equipment. VR technology is a comprehensive technology integrating computer technology, sensor technology, psychology, and physiology. The main simulation objects include environment, skills, and perceptions. It aims to simulate the external environment through the simulation software and hardware systems such as computers, audio-visual terminals, and tactile terminals and provide users with highly restorable, informative, and interactive virtual physiological and psychological experiences. AR technology is a combination of the virtual and the real. It simulates through computers and terminals such information as vision, sound, taste, and touch that are difficult to experience in a certain time and space. It then superimposes the information on the real world for human senses to recognize to achieve cognitive experience beyond reality.

Attention is the concentration and focus of people’s mental efforts—selective, transferable and distractible. Attention restoration is the process in which attention is distracted, briefly shifted and focused again (John, 2000). The attention restoration theory holds that in order to carry out daily life efficiently, people must keep their cognition clear, and clear cognition requires focused attention. The decline of focused attention ability will lead to many negative effects, such as reduced irritability, reduced planning ability, reduced sensitivity to interpersonal information, and increased cognitive error rate. However, focused attention requires individuals to ignore all potential distractions, which leads to huge energy consumption and fatigue (Stephen, 1995). Attention is a limited resource (Mihaly, 2009). The decline of attention will directly affect the teaching effect of ideological and political theory courses in colleges and universities. To achieve good effect in traditional teaching mode, teachers find it is a must to keep students fully aware of what is going on and frequently call their attention. At the same time, students are required to ignore all potential interference in class. This places a heavy burden on both teachers and students.

Ideological and political theory courses in colleges and universities, such as Basic Principles of Marxism, emphasize theoretical study. The teaching contents of these courses are often abstract, monotonous and theoretically challenging. Naturally, students may often find them too boring and difficult and burdensome since these courses usually require students to be good at abstract reasoning. So, the confusion, repetition and the complex learning content make it all the more difficult for students to restore attention. VR and AR technology is applied to teaching ideological and political theory courses in colleges and universities to solve this problem. This technology can improve the attraction of teaching contents, help to hold students’ attention, and promptly restore attention if diverted. The cutting-edge MR technology can combine the advantages of both VR and AR, help students to visualize what they study, and reduce their cognitive burden. In the VR and AR learning environment under the framework of immersive media technology, teachers will show the course contents to students through mental and perceptual simulation, create a virtual space conducive to students’ understanding and learning, or present the virtual contents in the real situation, to engage students in teaching through real-time interaction. When the learning situation ultimately attracts students’ senses, their consciousness will narrowly focus on learning activities. At the same time, the differences between themselves and the environment, between stimuli and reactions, between the past, present and the future will almost disappear. They will only respond to clear goals and specific feedback, and irrelevant perceptions and ideas will be filtered out. Another important factor that affects students’ attention restoration is the freedom learners enjoy thanks to the application of immersive technology. The greater the freedom is the easier it is for the students to ignore irrelevant information and focus on what they should learn.

**Figure 1:**
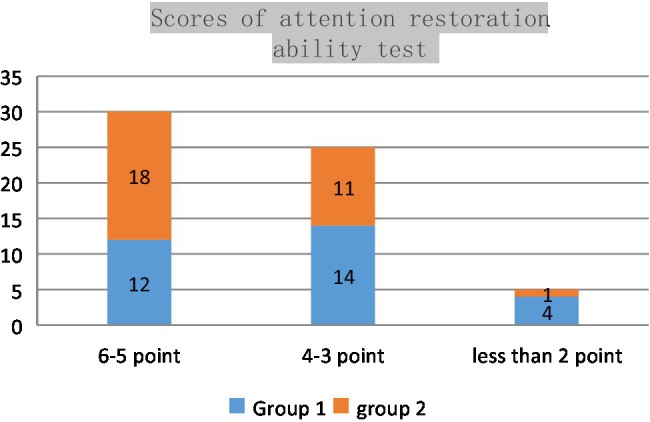
Scores of attention restoration ability test.

In the questionnaire, the students of Group 1, who only learned through watching the video, were compared with the subjects who learned through VR teaching media made with immersive media technology. Whenever a major battle named after a river appears in the course for the first time (the teaching contents for the two groups are basically the same. Six major battles were mentioned. Only the first time counted), the subject needs to raise his hand within 3 s. A correct move scores one point; a wrong or failed one scores none. The number of students in the second group with a score of 6-5 is significantly higher than that in the first group. It can be seen that the use of immersive media technology in ideological and political theory teaching can effectively improve students’ attention restoration ability (Figure 1).

### The Combination of Cognitive Experience and Emotional Experience Opens Up a Wider Learning Space

A learner’s learning experience mainly consists of cognitive experience and emotional experience. The change of cognitive experience causes emotional response, so the promotion of emotional experience is based on the promotion of cognitive experience (Elliott, 2018). Cognition is knowing, learning, and understanding based on perceptions and judgment. Emotional experience, on the other hand, is sympathetic response to familiar experiences and the culture and spirit conveyed by the media contents. The learning situation displayed by immersive media technology often features a clear visual and realistic cognitive experience environment, and an immediate feedback and emotional incentive mechanism, which facilitates students’ proper cognition, and arouse in them “a more sympathetic response, so that students may identify with happiness and sorrows in the situation, thus unconsciously informed and edified” (Suzhou Junior High School Chinese Teaching Panel, 2010).

**Figure 2:**
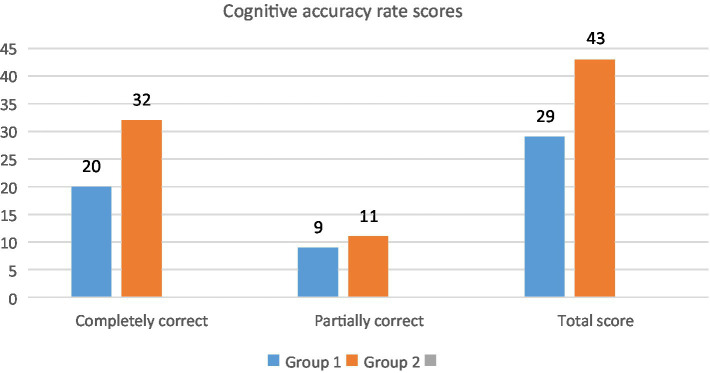
Cognitive accuracy rate scores.

One of the biggest limitations of traditional classroom teaching methods lies in the limitations of time and space on teaching situations. It is not easy to simulate and reproduce some specific historical scenes in an all-around way to teach ideological and political theory courses in colleges and universities in a traditional way. Due to various objective factors, some historical scenes and historical figures are displayed only by one-dimensional text descriptions or two-dimensional photos and videos in the teaching process, which are usually not easily understood and accepted by students (Chen, 2018; Elliott, 2018). With the help of AR and VR terminals under the framework of immersive media technology, we can create a specific learning situation that conforms to the teaching contents through the virtual stimulation of senses such as vision, hearing, and touch. The historical scenes and characters can be simulated sufficiently realistic. So, teachers can decide to give instructions any time they want, but students can also “experience” the moments of history through simulated scenes and human-computer interaction. This breaks the limitation of time and space of classroom teaching and narrows the gap between theory and historical facts. Its effects on students’ accurate understanding is shown in Figure 2.

The questionnaire had a task: match the following statements with the battles they describe—Crossing Dadu River/Crossing Xiangjiang River/Crossing Jinsha River. Statement A was “This is the most tragic and fierce war on Red Army’s Long March. The river is dyed red with blood.” Statement B was “For 7 days and nights, the main force of the Red Army only relied on seven boats to cross the river.” Statement C was “The Red Army soldiers carried muskets and few grenades, holding on to the rickety iron rope and braving the fierce fire on the other side.” Students would score 2 points if they gave all correct answers, 1 point for some correct answers and 0 point for all wrong answers. The second group using immersion media had obvious cognitive accuracy advantages compared to the first group using only text materials and static PPT slides. It can be seen that the use of immersive media in teaching can effectively improve the accuracy of understanding and enhance the level of cognitive experience.

Fostering patriotism and cultivating national confidence is one of the important teaching tasks of ideological and political theory courses in colleges and universities. Immersive media technology plays a positive role in creating emotional experiences and arousing emotional resonance. The most significant advantage of immersive media is to render the audience immersed. Real pictures, sounds, colors, shapes, etc., make the audience completely immersed in the “real world” of the virtual world and impact the emotional experience of the audience. Wise use of immersive media technology can enhance students’ emotional experience and effectively improve the teaching effect (Clifford and Montgomery, 2015). One of the important factors affecting students’ emotional experience is the vividness of learners’ experience in immersive media. Because students perceive things in the virtual learning environment the same as they do in the real world, they can feel the existence of “me” in the virtual world. This psychological identity makes learners feel keener about the experience. This emotional experience is positive and active, enabling learners to have a great sense of pleasure and fulfillment in the participation process, thus effectively improving the teaching effectiveness.

**Figure 3:**
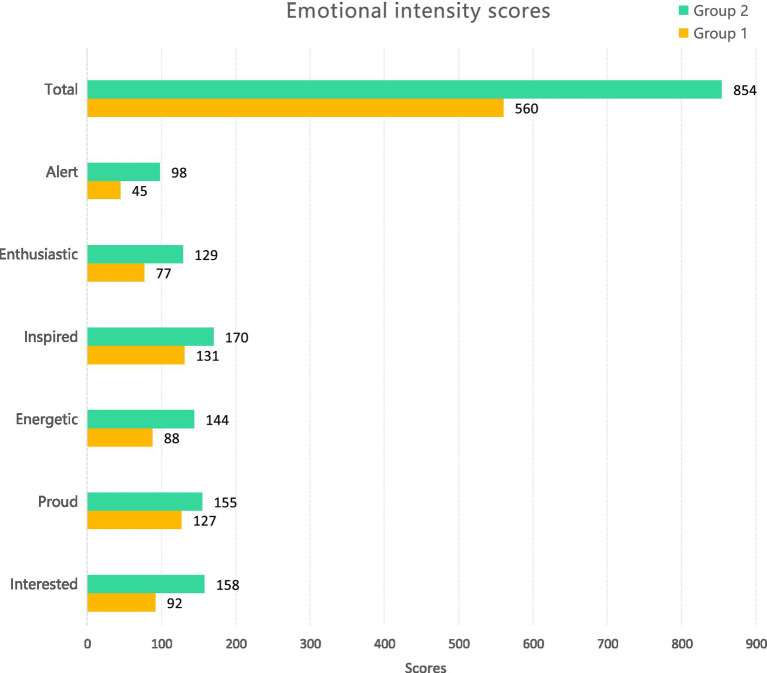
Emotional intensity scores.

The positive and negative affect scale (PA-NAS) compiled by Watson and others was used to test the degree of participation in the emotional experience of the two groups. In the questionnaire (as shown in Table 2), five ideological and political theory courses, teachers preset six indicators of emotional response expected of students in teaching practice: interested, enthusiastic, proud, inspired, energetic, and alert. In the experiment, the numbers 1–5 represent the emotional intensity, and the larger the number, the higher the emotional intensity. The data gathered from the questionnaire completed by the two groups reveal that the first group scored 854 points, and the second earned 560. See Figure 3 for statistics of specific emotional indicators. It can be seen that the application of immersion media enables the second group to have a significantly stronger intensity of all emotions listed than the first group, who were taught otherwise. It can be seen from the data in the table that there are significant differences between the two groups, both individually and collectively. Therefore, compared with traditional teaching media, immersion teaching media tend to arouse stronger feelings.

**Table 2:** Students’ self-assessment of ability improvement.

	Cooperation	Ability to handle different situations	Thinking ability	Esthetic understanding	Other abilities
Group 1	0	0	5	0	1
Group 2	7	1	14	12	7

Compared with traditional media, immersive media technology applied to teaching ideological and political theory courses in colleges and universities can give full play to the advantages of this technology in enhancing cognitive experience and emotional experience, providing students with a superior learning experience significantly improve teaching effectiveness.

### The Integration of Human–Computer Interaction and Interpersonal Interaction Enriches the Growth Environment of Subjects

The communication process of immersive media is “people-centered information communication, the blend of human nature and personality” and “a process of subjective creation based on the objective” (Li, 2017). The subjects who used immersive media to learn ideological and political theory courses are college students. To give full play to the advantages of immersive media in improving teaching effectiveness, they have to be learner-centered, which means they should accommodate students’ personality and help with the growth of students’ abilities. The process of teaching is itself a process of communication between teachers and students and development on both parts. Without communication and interaction, teaching will not exist or it will be difficult to teach” (Guo, 2006). Immersive media technology can provide an environment of human-computer interaction and interpersonal interaction, including “social integration, creating new experiences” and “providing special incentive mechanism” to support the cooperative learning model, promote students’ self-affirmation and self-harmony, and at the same time, help them to realize the growth of cooperation and communication skills, which is an essential advantage that the traditional teaching model does not have (Wang, 2016).

Immersive media can provide a human-computer interaction beyond the traditional sense. Relying on the interactive sensing function, the immersive media software and hardware can create a virtual environment or a physical sensory experience combining the real and the virtual. In this interactive environment, learners emotionally communicate with the virtual world and become doers in the experience. They perceive and behave. Instead of simply receiving information and various sensory stimuli in immersive media, they actively participate in the construction and presentation of the virtual world. In the brand-new “human-computer interaction” mode, they can feel the three-dimensional and all-round environmental stimuli like they do in the real world and enjoy the self-harmony attributable to the scientific mechanism of progressive learning and the challenge of teaching games that match their skills. This self-harmony is a kind of “advanced human experience, which leads to development, self-realization and personality perfection” (Vasilyuk, 1989). In this active learning state, which is caused by the deep interaction between people and devices, students may complete some learning tasks that cannot be fulfilled under normal conditions, and achieve learning effects beyond expectations. However, learners often do not realize that the challenges brought by learning activities have already exceeded the level that they can handle in the past. In this case, students will fully affirm their own learning ability and be more willing to study hard for new knowledge to achieve a positive state of pleasure, satisfaction, excitement, and fulfillment. They will be inspired to pursue growth on a higher and more complex level.

The cultivation of team spirit and communicative skills is generally considered to be an essential part of building up moral integrity and cultivating people in colleges and universities. In this regard, the most effective way of education is to complete practical tasks through communication and collaboration with others in real situations, following the learning method of social construction. The virtual environment created by immersive media technology enables “people to communicate with each other through virtual reality devices to the effect that they have a face-to-face communication” (Wang, 2016). Students can practice and test their learning achievements through human-computer interaction and peer interaction in virtual situations, and even get the experience of solving practical problems with learning partners in scenes they are unlikely to witness in real life (because of cost, danger, contingency, and other factors), so as to improve their comprehensive ability (Zhang, 2020).

**Figure 4:**
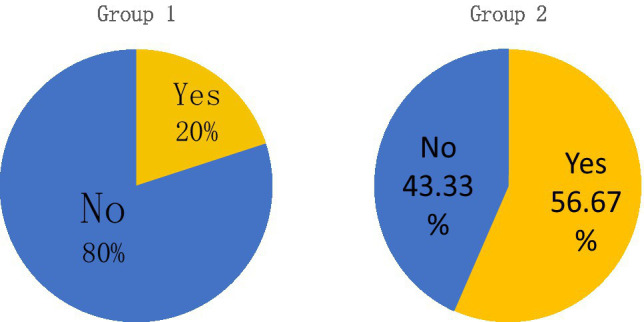
Statistics of self-assessment ratio of students’ comprehensive ability improvement.

Group 1, which only learned about the Battle of the Long March on the Jinsha River through traditional classroom teaching media, was compared with the subjects who studied through the VR interactive simulation experience system “Crossing Jinsha River” made with immersive media technology. The questionnaire asked this question: Through this round of learning, do you feel that you have gained and improved in other abilities besides classroom knowledge? If yes, please describe it.” The responses given by the two groups are shown in Figure 4 and Table 3. A significantly higher number of students in Group 2 believed they improved their ability in other respects. These students think their improvement lie in thinking ability, collaboration, ability to handle different situations, esthetic understanding and other aspects, while those who have the same opinion in Group 1 tend to believe their improvement only lies in thinking ability. It can be seen that, in teaching ideological and political theory courses in colleges and universities, giving full play to the interactive advantages of immersive media technology will effectively stimulate students’ learning motivation, promote the growth of students’ comprehensive ability, and attain some of the goals of building up moral integrity and cultivating talents.

**Table 3:** Questionnaire for emotional responses.

Please tick √ your emotional intensity after the learning process is completed (please tick one for each and every emotional response).					
Types of emotions	Strength 1 point	Strength 2 points	Strength 3 points	Strength 4 points	Strength 5 points
Alert	ÿ	ÿ	ÿ	ÿ	ÿ
Enthusiastic	ÿ	ÿ	ÿ	ÿ	ÿ
Inspired	ÿ	ÿ	ÿ	ÿ	ÿ
Energetic	ÿ	ÿ	ÿ	ÿ	ÿ
Proud	ÿ	ÿ	ÿ	ÿ	ÿ
Interested	ÿ	ÿ	ÿ	ÿ	ÿ

## Ways to Improve the Teaching Effectiveness of Ideological and Political Theory Courses in Colleges and Universities Based on Immersive Media Technology

The increased use of immersive media technology in the field of education means an ever-growing impact on the improvement of teaching effectiveness. However, in order to rapidly improve the teaching effectiveness of ideological and political theory courses in colleges and universities, we should explore the relationship between the teaching of these courses and immersive media technology from the perspective of theory and practice, and seek for a new way to improve the teaching effect of ideological and political theory courses in colleges and universities through the application of this technology.

### The Well-Designed Narration Makes the Theoretical Learning Model Leap From “Rational Cognition” to “Affective Identification”

Affection is a person’s attitude toward the relationship between objective things and himself. Affective identification refers to a psychological process when people have positive feelings for others, groups or things, and then emulate or assimilate them as the driving force to achieve their own goals. It is different from perceptual cognition. The realization of affective identification has to be based on reason. If a person cannot identify with another person or thing in terms of ideas, s/he is unlikely to develop a positive attitude of closeness, admiration, or trust emotionally. It can be said that the degree of affective identification indicates the degree of internalization of people or things that people pay attention to.

For students, in learning ideological and political theory courses in colleges and universities, affective identification has the functions of selection, motivation and correction. First of all, it can induce positive emotions to influence the judgment standard of students when they recognize things. Whether college students can resonate with the ideological and political theory courses will inevitably depend on the individual’s emotional choice. Successful stimulation of students’ inner positive emotions will make them actively participate in the learning process, thus resulting in an improved teaching effect. Secondly, effective identification is also the driving force for people to realize their value. With it, they will be motivated consciously or unconsciously into action. If students are expected to internalize the teaching content of the ideological and political theory course and externalize it into practice, they must have positive emotional experience first. Thirdly, effective identification has a curative effect. It can eliminate destructive emotions and improve people’s willpower and self-control. Students will inevitably encounter fatigue, difficulties and problems in the learning process. Cultivating their affective identification with the courses and the curriculum contents can ensure that they learn with a healthy, stable and positive attitude.

At present, the actual situation is that college students’ understanding of ideological and political theory courses basically stays at the level of “rational cognition.” To improve the teaching effectiveness, they have to leap from “rational cognition” to “affective identification.” The starting point to achieve this goal lies in the narrative way of teaching. The narrative is an essential means of emotional motivation and knowledge transmission, whatever teaching methods are concerned. The ideological and political theory courses are highly theoretical, abstract, and challenging. Still, they have a close connection with social life, so we should pay more attention to the innovative narrative methods in the teaching process. Immersive media has the unique advantages of breaking the limitation of time and space, vividly representing historical situations, turning abstract into concrete and arousing emotional resonance. Suppose we can creatively use immersive media technology in the narration while teaching these courses. In that case, we can create a teaching situation that enables students to appreciate the historical situation of the times fully, clearly understand the theoretical logic of the teaching content, deeply experience the relationship between the course content and their life practice, and cultivate their emotional identification with the Communist Party of China, our country and the socialist system with Chinese characteristics. The integration of immersive media technology and educational informatization undoubtedly provides an all-around upgrade for the narrative ability of ideological and political theory teaching in colleges and universities. Driven by the fascinating narrative carefully designed with the support of VR and AR technology, students can fully understand and internalize what they have learned into effective identification, and most willingly immerse themselves in the whole learning environment.

### Building a Virtual Teaching Platform That Can Enable Students to Have a “Time Travel Through History” Rather Than Just “a Glimpse of History” in History Classes

Historical knowledge is an essential part of the teaching content of ideological and political theory courses in colleges and universities. Teaching historical knowledge is one of the major tasks of teachers. For example, the course “Outline of Modern and Contemporary Chinese History” informs students of the basic theory, ideals and beliefs of Marxism by teaching modern Chinese history and guiding students to establish a correct world outlook, outlook on life, and values and scientific outlook on history. Another example is “Introduction to Mao Zedong Thought and the Theoretical System of Socialism with Chinese Characteristics.” This course is about the historical process of the CPC’s combination of the basic principles of Marxism with China’s reality. Even a course that focuses on a theoretical study such as “Basic Principles of Marxism” also includes historical materialism and other knowledge related to history.

The traditional way of teaching historical knowledge can only give students a glimpse of history. Teachers usually prioritize lecturing, supplemented by conventional teaching media such as historical documents, photos, audio, videos, and charts. They try to help students to understand and appreciate historical knowledge by showing them some representative sketches, clips and moments in the historical process. Under this teaching model, the historical images presented in students’ minds are not directly formed through visual and auditory perception as in the real world. They are just some mental pictures which are spliced by the refraction of their own experience and imagination. These pictures are not three-dimensional or clear, so they are unlikely to arouse students’ emotional identification.

The application of immersive media technology in practice has remarkably influenced the teaching of ideological and political theory courses in colleges and universities. Although it will not completely replace the traditional teaching mode, it is sure to play a significant and positive role in constructing knowledge structure and improving efficiency and depth of knowledge transmission, especially when it comes to historical knowledge. By means of virtual reality and augmented reality, students are encouraged to apply what they have learned to lifelike or simulated historical or social contexts. This becomes the main contents and way of teaching. The combination of learning with practice enables students to immerse themselves in specific situations and take initiatives to learn. Undoubtedly, the advantage of immersive media in the teaching of history knowledge is obvious. By creating a virtual world, restoring a specific historical situation, and promoting the understanding of historical knowledge in the ideological and political theory course through the integrated experience of multiple senses, such as vision, hearing, touch and smell, immersive media can effectively help teachers and students to overcome learning difficulties and appreciate the times, and improve the teaching effectiveness by controlling the sense of presence in the teaching process. The application of this technology to these courses can provide students with an excellent learning experience similar to “time travel through history.” However, colleges and universities must build a special virtual simulation teaching platform to support the teaching of ideological and political theory courses if they want to scale up and sustain the excellent teaching effectiveness brought by this teaching method based on advanced technology.

The virtual simulation teaching platform based on immersive media technology can enable students to be immersed in historical scenes from the first person’s perspective and devote themselves fully to learning. The design of learning content, based on the teaching materials of ideological and political theory courses, covers representative historical knowledge points in key chapters, and fully combines the advantages of red cultural resources in the region where the university is located to create an immersive media learning situation with strong sense of participation, immersion, ceremony, experience and interaction. In terms of curriculum arrangement, the class hours completed in the virtual simulation teaching platform can account for a part of the whole class hours, and the performance in the learning process is included in the class grades. When learning on the virtual simulation platform, students can have the right to freely combine relevant learning modules based on their own interests and command of knowledge and decide what to learn first to enjoy maximum autonomy in learning. This will be followed by improved teaching effectiveness of history knowledge.

For teachers, the strengths of immersive media technology lie in its duplicability and safety. In teaching, learners can be placed in various situations that they are unlikely to encounter in reality to carry out targeted training on their own for as many times as they want, so as to give full play to students’ subjectivity, maintain their interest in learning and quickly improve their command of knowledge and skills. In addition, safety is the most critical consideration in practical teaching. The potential danger faced by the virtual environment is far less than that in reality. Learners can boldly try various learning options in a virtual environment. Even if serious mistakes occur, they will not cause the same adverse consequences and damages as in the real world. At the same time, learners’ error rate, learning time and other data will also be recorded and evaluated by the platform as the reference for the final assessment.

### Human–Computer Interaction Transforms the Way of Teaching From “Copying Ideas” to “Exploring Meaning”

At present, teachers usually try to imbue students with different ideas by teaching ideological and political theory courses in colleges and universities. The conventional indoctrination way is questioned with the connotative development of education and teaching reform of ideological and political theory courses. In essence, indoctrination is an effective way to turn ideas into power. Indoctrination in teaching ideological and political theory courses is the only way for political parties to occupy ideological vantage. It is also inevitable for Chinese universities to adhere to the socialist direction of running schools. No matter how innovative the teaching methods are, the ideological and political theory courses must stick to the correct political status and value orientation in the new historical era. The basic principles of indoctrination remain unchanged, but the methods of indoctrination can be changed. Indoctrination in teaching ideological and political theory courses in colleges and universities does not mean that students are required to “copy and paste” the teaching materials and immediately accept and follow what is taught. In fact, in order to truly realize the teaching task of building up moral integrity and cultivating talents of virtues, colleges and universities must adhere to the heuristic methods in teaching ideological and political theory courses, consciously follow the law of cognition and the law of physical and mental growth, and teach these courses scientifically and effectively.

Heuristic teaching conforms to teaching objectives and objective laws of learning. It also takes into consideration students’ natural conditions. Basically, it refers to “using such teaching methods as elicitation and heuristic teaching to impart knowledge and cultivate abilities, so as to urge students to study on their initiative, and thus to promote their physical and mental development” (Guo, 2006). In order to solve the problems existing in the teaching of ideological and political theory courses in colleges and universities, the application of immersive media technology into the teaching process can give full play to the advantages of the technology in human-computer interaction, combine indoctrination with elicitation and heuristic teaching, and improve the teaching effectiveness. Setting proper problem-solving situations is crucial to the success of heuristic teaching. Immersive media technology can provide interactive and controllable learning situations for students. In these situations, students will take the initiative to think and explore rather than passively receiving or mechanically copying the ideas fed to them. The basis of heuristic teaching is to stimulate students’ inner learning motivation. In the virtual environment based on immersive media technology, VR and AR technology can mobilize students’ sensory perception, guide their psychological feelings, and create an overall atmosphere to stimulate their interest and initiative and maximize their participation in learning activities. Heuristic teaching emphasizes the connection between theory and practice and the combination between knowledge and direct experience (Skedsmo and Huber, 2018). Immersive media technology can provide vivid and visual thinking materials for students’ learning and understanding activities. The human-computer interaction breaks through the limitation of time and space on teaching activities so that students can participate in teaching activities in realistic VR environments. It helps to strengthen their cognition of abstract concepts and supernatural phenomena, and guide them to further explore and think about the intrinsic meaning of what they have learned.

When students are reasoned into the conviction of the political views, they will learn to internalize the views into their ideals and beliefs. They will be more likely to be edified by noble sentiments and guide their behavior with sound values, thereby enhancing the teaching effectiveness of ideological and political theory courses.

## Conclusion

Immersive media technology integrates state-of-the-art technologies in such fields as psychology, acoustics, optics, and computer science. It can improve teaching effectiveness and promote innovative teaching ideas and methods when applied to the teaching of ideological and political theory courses in colleges and universities. In immersive-media-technology-aided education, teachers of these courses should accommodate the psychological features and needs of college students as well as the teaching contents and plans. Based on these considerations, they should also adopt new teaching tools such as human-computer interaction, virtual environment reproduction, and augmented reality experience. This research has proved that the courses’ purpose of building up moral integrity and cultivating talents of virtues will be better achieved if the advantages of immersive media technology in improving attention restoration, practical ability and emotional and cognitive experience can be brought into full play. Moreover, the application of immersive media technology will also produce a positive spillover effect. It is conducive to the improvement of college students’ communicative skills, cooperation ability, esthetic ability and physical and mental wellbeing.

## Data Availability Statement

The raw data supporting the conclusions of this article will be made available by the authors, without undue reservation.

## Ethics Statement

Ethical review and approval was not required for the study on human participants in accordance with the local legislation and institutional requirements. Written informed consent from the patients/participants was not required to participate in this study in accordance with the national legislation and the institutional requirements.

## Author Contributions

LS is the major contributors of this work, contributed to the conception of the study and performed the data analyses and wrote the manuscript; ML contributed significantly to analysis and manuscript preparation, helped perform the analysis with constructive discussions.

## Funding

This work was supported by the Youth Research Project National Social Science Fund of China “A Study of General Secretary Xi Jinping’s Thought on ‘Mind’ Study of Communists” (18CKS034).

## Conflict of Interest

The authors declare that the research was conducted in the absence of any commercial or financial relationships that could be construed as a potential conflict of interest.

## Publisher’s Note

All claims expressed in this article are solely those of the authors and do not necessarily represent those of their affiliated organizations, or those of the publisher, the editors and the reviewers. Any product that may be evaluated in this article, or claim that may be made by its manufacturer, is not guaranteed or endorsed by the publisher.
